# Rice Bran Ash Mineral Extract Increases Pigmentation through the p-ERK Pathway in Zebrafish (*Danio rerio*)

**DOI:** 10.3390/ijms20092172

**Published:** 2019-05-02

**Authors:** Yu-Mi Kim, Eun-Cheol Lee, Han-Moi Lim, Young-Kwon Seo

**Affiliations:** Department of Medical Biotechnology (BK21 Plus Team), Dongguk University, Goyang-si 10326, Korea; kjmtik@nate.com (Y.-M.K.); eunchul90@gmail.com (E.-C.L.); gksahl321@gmail.com (H.-M.L.)

**Keywords:** pigmentation, melanogenesis, zebrafish, p-ERK signaling pathway

## Abstract

The purpose of the present study is to evaluate the effect of rice bran ash mineral extract (RBM) on pigmentation in zebrafish (*Danio rerio*). Melanin has the ability to block ultraviolet (UV) radiation and scavenge free oxygen radicals, thus protecting the skin from their harmful effects. Agents that increase melanin synthesis in melanocytes may reduce the risk of photodamage and skin cancer. The present study investigates the effect of RBM on pigmentation in zebrafish and the underlying mechanism. RBM was found to significantly increase the expression of microphthalmia-associated transcription factor (MITF), a key transcription factor involved in melanin production. RBM also suppressed the phosphorylation of extracellular signal-regulated kinase (ERK), which negatively regulates zebrafish pigmentation. Together, these results suggest that RBM promotes melanin biosynthesis in zebrafish.

## 1. Introduction

Melanin is a brown-black pigment principally responsible for the color of skin, hair and eyes [1] and has various functions including the protection of the skin from ultraviolet (UV) light and the removal of reactive oxygen species. Thus, melanin is important for skin homeostasis, and tanning represents a distress signal [1,2]. Epidermal melanocytes synthesize melanin pigments and play an important role in skin pigmentation by absorbing UV radiation, thereby serving as a natural sunscreen [3]. In addition, UV absorption by the skin not only triggers mechanisms that defend skin integrity and regulate global homeostasis but also induces skin pathology (e.g., cancer, aging and autoimmune responses) [4]. Therefore, there is increasing focus on identifying regulators of the melanogenesis process to develop novel treatments for depigmented skin disorders.

Many studies have shown that a wide variety of natural extracts promote melanogenesis. Hong et al. reported that *Rhynchosia nulubilis* and *Rhynchosia volubilis* ethanol extracts promote melanin synthesis by increasing tyrosinase activity [5]. Studies have also shown that lemon balm extract promotes melanogenesis in melanoma cells [6]. In addition, a variety of other natural extracts for increasing pigmentation have been studied, including *Cornus officinalis* methanol extract [7], *Ardisia crenata* extract [8], and *Zingiber cassumunar Roxb.* [9]. Similarly, many other studies have used organic materials to stimulate pigmentation.

In contrast, few studies have evaluated inorganic mineral components’ ability to stimulate pigmentation. In our previous study, we demonstrated that rice bran ash mineral extract (RBM) promotes melanogenesis via phosphorylation of 3′,5′-cyclic adenosine monophosphate (cAMP)-responsive element binding protein (CREB) in human melanocyte cells and in a three-dimensional human hair dermal papilla-melanocyte co-culture model [10,11]. Rice bran ash, which is produced by incinerating rice bran, is rich in silicon (Si, 65 wt%) and contains other minerals including potassium, carbon, phosphorus, MgO, CaO, Na_2_O, Al_2_O_3_, ZnO, K_2_O, MnO_2_, and Fe_2_O_3_, as well as soluble silicic acids and organic compounds [10,12].

A new in vivo pigmentation model using zebrafish has been recently developed. The application of this model for studying the pigmentation activity of many bioactive compounds has received significant attention in medicine, genetics, chemistry, and cosmetics [13,14]. Zebrafish have many advantages including a large number of offspring per generation, small size, easy maintenance and handling [15], and a high efficiency of drug penetration through the skin and gills; furthermore, zebrafish contain melanin pigment on their body surface, facilitating the observation and evaluation of pigmentation-related processes [16,17]. In the present study, we studied the in vivo stimulatory effects of RBM on pigmentation in zebrafish. To assess changes in pigmentation, we performed melanin content assays, Western blot analysis, and Fontana-Masson staining. At the mechanistic level, we analyzed changes in extracellular signal-regulated kinase (ERK) phosphorylation signaling associated with microphthalmia-associated transcription factor (MITF) regulation.

## 2. Results

### 2.1. Reverse Transcriptase-Polymerase Chain Reaction (RT-PCR)

**Figure 1:**
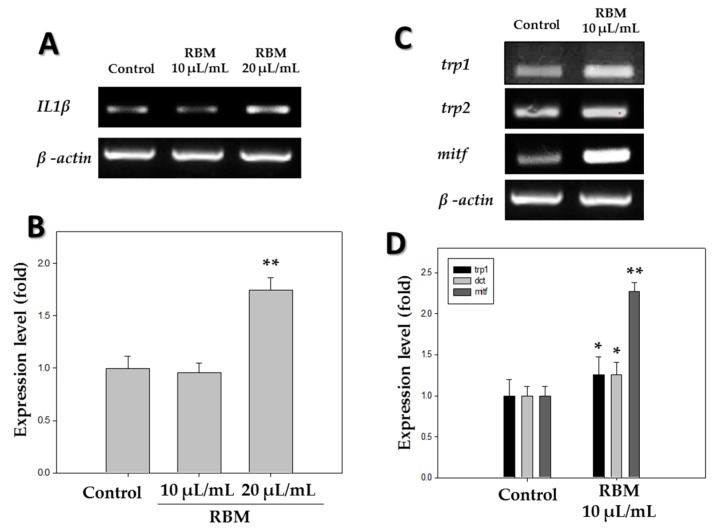
Results of mRNA expression analysis using reverse transcription-polymerase chain reaction in zebrafish (*n* = 15) after treatment with rice bran mineral extract (RBM) at 5 days post-fertilization (dpf). (**A**) mRNA expression of the pro-inflammatory cytokine *IL1β*; (**B**) Expression level of *IL1β* mRNA using *β-actin* as the reference gene; (**C**) mRNA expression of pigmentation-related genes; (**D**) Expression levels of *trp1*, *trp2*, and *mitf* using *β-actin* as the reference gene. * *p* < 0.05, ** *p* < 0.01, compared to control.

The mRNA expression levels of pigmentation-related genes and inflammation-related genes in zebrafish larvae were measured at 5 days post-fertilization (dpf). As shown in Figure 1A,B, the pro-inflammatory cytokine *IL1β* was significantly induced with 20 µL/mL RBM treatment compared to the control group, while the level of induction was insignificant at 10 µL/mL. The mRNA levels of tyrosinase-related protein-1 (*trp1*) and tyrosinase-related protein-2 (*trp2*) in the zebrafish treated with RBM were an average of 1.2-fold higher than those in the control group, and the *mitf* mRNA level was 2.2-fold higher upon RBM treatment compared with that of the control group (Figure 1C,D). These data demonstrate that RBM increased pigmentation-related gene expression in zebrafish.

### 2.2. Melanin Assay

**Figure 2:**
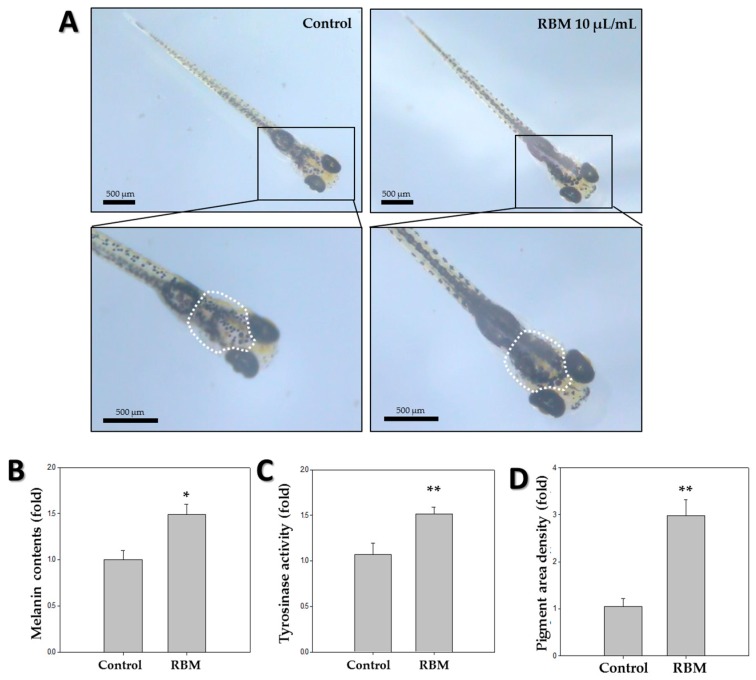
Effects of rice bran ash mineral extract (RBM) on pigmentation in zebrafish. (**A**) Synchronized zebrafish embryos (*n* = 20) were exposed to RBM, and pigmentation was observed under a stereomicroscope via inferior views of the embryos at 5 dpf; (**B**) Zebrafish melanin content was detected by melanin content assay at 5 dpf (*n* = 80); (**C**) Tyrosinase activity in zebrafish (*n* = 80) was detected by tyrosinase assay at 5 dpf; (**D**) Pigmentation density in the indicated white-outlined area was measured in treated embryos and normalized to that of control embryos using the software ImageJ. * *p* < 0.05, ** *p* < 0.01, compared to control. Scale bar; 500 µm.

To measure the effect of RBM on pigmentation in zebrafish, melanin levels were determined using the melanin content assay. Compared to the control group, the melanin content of zebrafish treated with RBM was approximately 1.5-fold greater (Figure 2B). These results indicate that RBM increased zebrafish pigmentation.

### 2.3. Pigmentation

To further measure pigmentation in vivo, a region of interest was selected and marked with a white contour, covering the dorsal pigment spot beginning midway between the eyes and extending around the pigmented eyes to the base of the head (Figure 2A). The density of the pigmented area in the treated embryos was normalized to that in the control embryos using ImageJ. The analysis shows that RBM treatment increased skin melanin formation by more than threefold in the zebrafish larvae at 5 dpf (Figure 2D).

### 2.4. Tyrosinase Assay

Tyrosinase is the most important enzyme in melanin biosynthesis. Therefore, zebrafish tyrosinase activity assay was conducted by measuring 3,4,-Dihydroxyphenylalanine (DOPA) oxidase activity, and the results are shown in Figure 2C. Compared to the control group, zebrafish treated with RBM showed ~1.5-fold increased tyrosinase activity (Figure 2C). These results indicate that RBM increased tyrosinase activity in zebrafish.

### 2.5. Western Blotting

**Figure 3:**
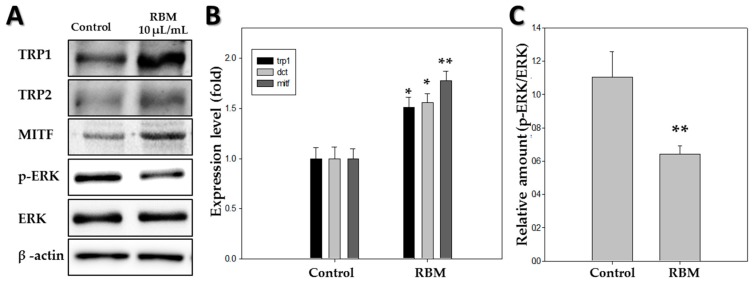
Effect of rice bran ash mineral extract (RBM) on protein levels of tyrosinase-related protein 1 (TRP1), microphthalmia-associated transcription factor (MITF), tyrosinase-related protein 2 (TRP2), and β-actin in zebrafish (*n* = 30) at 5 dpf. (**A**) Western blotting analysis of pigmentation-related proteins; (**B**) Expression levels of TRP1, TRP2, and MITF; (**C**) Level of p-ERK (extracellular signal-regulated kinase). Each bar represents the mean ± standard error of independent experiments performed in triplicate (*n* = 5). (* *p* < 0.05, ** *p* < 0.01, compared to control).

Expression of pigmentation-related proteins in 5 dpf zebrafish was measured using western blot analysis after treatment with 10 µL/mL RBM. As shown in Figure 3A,B, the expression of all pigmentation-related proteins, including tyrosinase-related protein 1 (TRP1), TRP2, and MITF increased by at least 1.5-fold compared to that in the control group. To assess the signaling pathways involved, we analyzed the phosphorylation of ERK (Figure 3A,C). In zebrafish treated with RBM, ERK phosphorylation was significantly decreased. Thus, western blotting analysis demonstrated that RBM treatment stimulated pigmentation in zebrafish.

### 2.6. Fontana-Masson Staining

**Figure 4:**
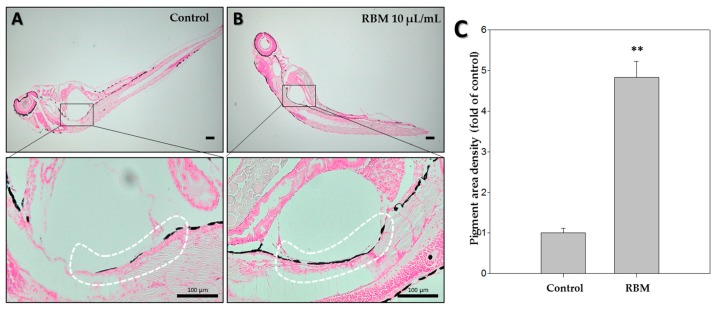
Effects of rice bran ash mineral extract (RBM) on zebrafish at 5dpf. Representative images of Fontana-Masson-stained zebrafish (dark color indicates secreted melanin). (**A**) Control; (**B**) RBM; (**C**) The pigmented area density in (**A**,**B**) was normalized to that of the control using ImageJ software. ** *p* < 0.05, compared to control. Original magnification: ×40 and ×100; scale bar, 100 µm.

To visualize melanin content in vivo, zebrafish larvae were subjected to Fontana-Masson staining, a common method used to assess melanin synthesis. A region of interest was selected to measure the pigmentation area, as indicated by a white outline. As shown in Figure 4, the staining of melanin granules (black color) was significantly increased with RBM exposure compared to the controls.

## 3. Discussion

Melanin is an effective absorber of light, capable of protecting human skin from damage caused by UV radiation [18]. Melanogenesis plays a critical role in protecting skin against external stresses such as UV irradiation and oxidative stressors [19].

The human skin contains a defensive melanocortin system to neutralize ultraviolet radiation consisting of the pigment melanin and its associated cleaved proopiomelanocortin (POMC) peptides. In melanocytes, ultraviolet radiation stimulates POMC formation, with consequent release of various POMC peptides. As a result, melanocyte-stimulating hormones and corticotropin are produced, which serve to regulate melanogenesis. POMC undergoes post-translational cleavage to produce its melanocyte-stimulating form that binds to the melanocortin 1 receptor on melanocytes, resulting in cAMP upregulation. Elevated cAMP stimulates expression of MITF, which then regulates the transcription of pigmentation enzymes, including tyrosinase, TRP1 and TRP2. As such, melanin biosynthesis is a complex and tightly regulated process [1,20,21,22].

Zebrafish is a popular vertebrate model organism with organ systems and gene sequences similar to humans [23]. Zebrafish have been established as a new in vivo model to evaluate the pigmentation activity of melanogenic regulatory compounds [24]; zebrafish embryos can be obtained in large numbers and are sufficiently small to facilitate easy handling and observation [25].

Rice bran ash, produced by incinerating rice bran, is rich in silicon and contains other minerals including potassium, carbon, and phosphorus [12,26]. In our previous study, we demonstrated that RBM comprising silicic acid promotes pigmentation in human melanocyte cells and in a three-dimensional human hair dermal papilla-melanocyte co-culture model [10,11]. In the present study, we investigated the effect of RBM on zebrafish pigmentation.

We first examined the inflammatory response of RBM by assessing the expression of inflammation-related cytokines. After treatment with 20 µL/mL RBM, zebrafish larvae showed high induction of the pro-inflammatory cytokine *IL1β*, whereas no significant inflammatory response was observed in zebrafish at a concentration of 10 µL/mL RBM (Figure 1A,B). Therefore, subsequent in vivo pigmentation studies were conducted at a concentration of 10 µL/mL. The pigmentation effect of RBM on zebrafish was observed under a stereomicroscope in 5 dpf larvae (Figure 2A), and pigmentation area density was normalized to that in the control larvae (Figure 2D). As shown in Figure 2, we found that exposure to RBM increased pigmentation even at a low dose in 5 dpf larvae, without observable toxicity.

Next, we performed melanin content assays to assess melanin levels upon RBM treatment (Figure 2B). Melanin levels increased approximately 1.4-fold in zebrafish treated with RBM. To visualize melanin, we conducted Fontana-Masson staining (Figure 4A,B) and determined the pigmentation area density (Figure 4C). Compared with that in the control group, the extent of the pigmentation area was significantly increased in the RBM-treated group (black color). These in vivo results are consistent with our previous in vitro studies using RBM [10,11].

To assess tyrosinase activity, we performed tyrosinase activity assays by measuring DOPA oxidase activity (Figure 2C). Melanogenesis is regulated by various melanogenesis-related enzymes, among which tyrosinase is the most important [1]. In the melanogenic pathway, l-DOPA is oxidized by tyrosinase to dopaquinone, an intermediate that is common to both eu- and pheo-melanogenic pathways. l-tyrosine and l-DOPA are recognized as the consecutive substrates and intermediates of melanogenesis and increased supply of l-tyrosine leads only to increased melanin pigmentation. [27]. As shown in Figure 2C, RBM was more efficient at catalyzing melanin synthesis compared to the control group.

Pigmentation is regulated by several important enzymes, including TRP1 and TRP2 [28]. Expression of pigmentation-related genes such as tyrosinase, TRP1, and TRP2 is mediated by MITF [19,29]. MITF is well known as the master regulator of melanin synthesis [18,30] and controls several key mechanisms including pigmentation, dendricity, and proliferation in response to environmental factors such as UV radiation [31].

In the present study, expression levels of the above enzymes and transcription factor were determined using reverse transcriptase-polymerase chain reaction (RT-PCR) and western blot analysis. The results showed that treatment with RBM induced the expression of TRP1, TRP2, and MITF (Figure 1D and Figure 3B). In addition, we found that the pigmentation effects of RBM in zebrafish were mediated by MITF upregulation via activation of ERK mitogen-activated protein kinase signaling. Many pigmentation studies have shown that MITF is regulated by various components of the mitogen-activated protein kinase signaling pathway, including ERK [15,32,33], and that phosphorylation of ERK can downregulate MITF expression [8,24]. Our previous studies demonstrated that the ERK signaling pathway plays an important role in promoting pigmentation in zebrafish [34]. Therefore, western blot analysis was performed to identify the signaling pathway associated with the increased melanin synthesis caused by RBM treatment (Figure 3A,C). The results showed a significant decrease in ERK phosphorylation in the RBM-treated experimental group, which may be responsible for stimulating the pigmentation pathway via MITF activation (Figure 3). The above results demonstrated that RBM induced pigmentation by increasing expression of the *mitf* gene, which led to tyrosinase, TRP1, and TRP2 upregulation via the ERK signaling pathway. Thus, the present study shows that RBM can stimulate pigmentation in vivo.

## 4. Materials and Methods

### 4.1. Rice Bran Ash Mineral Extract

RBM was produced using a previously described method [11]. Briefly, RBM was obtained from the carbonized chaff of rice bran or rice bran ash (*Japonica*, *Oryza sativa*). Rice bran ash (200 g) was added to 1 L of distilled water and stirred at 100 °C. This mixture was centrifuged to remove remaining particulate matter, and the supernatant was filtered through 10 µm filter paper and sterilized by filtration through a 0.2 µm syringe filter. The chemical composition of the RBM is shown in Table 1.

### 4.2. Zebrafish Maintenance and Embryo Collection

All animal studies were approved by the Animal Care and Use Committee of Dongguk University (IACUC-2018-003-1, 4 May 2018), and all studies were performed under 2-phenoxyethanol anesthesia. Zebrafish were obtained from a commercial dealer, housed under a 14 h light/10 h dark cycle at 28.5 °C, and raised in water containing 1% sea salt. Embryos were obtained via natural spawning. Zebrafish larval developmental stages were represented as days post-fertilization (dpf). RBM was added to the zebrafish embryo medium, and the effects on zebrafish pigmentation were observed under a stereomicroscope. All evaluations of pigmentation were performed at 5 dpf.

### 4.3. Microscopy

To assess the pigmentation effect of RBM, embryos in the RBM-treated group and those in the untreated control group were observed and photographed under light microscopy at 5 dpf.

### 4.4. Melanin Content Assay

Zebrafish melanin content was evaluated following the method described previously, with slight modifications [35]. In brief, extracts of 5 dpf larvae were prepared as follows. The larvae were collected and lysed in 10% DMSO dissolved in 1 N NaOH and incubated at 100 °C for 1 h. The optical density of the supernatant was measured at 400 nm. Results from the treated embryos were compared to those of the appropriate controls, and the final measurements were reported as the fold change from the control group measurements.

### 4.5. Tyrosinase Assay

Zebrafish tyrosinase activity assay was conducted by measuring DOPA oxidase activity as previously described [23]. Treated embryos were washed with phosphate buffered saline (pH 6.8) and lysed with 1% (*v*/*v*) Triton X-100. The embryos were then disrupted by a freeze-thaw cycle, and lysates were collected by centrifugation at 15,000 rpm for 10 min at 4 °C. We performed a bicinchoninic acid assay to measure protein levels, using bovine serum albumin as a standard, adjusted to the same concentration as the sample protein with lysis buffer. A total of 90 µL/sample was loaded in each well of a 96-well plate, and 10 µL of 5 mM l-DOPA was added to all wells. After 30 min incubation at 37 °C, absorbance was measured at 475 nm. Results from the treated embryos were compared to those of the controls, and the final measurements were reported as the fold change from the control group measurements.

### 4.6. RT-PCR Analysis

Total zebrafish RNA was isolated using TRIzol (Invitrogen, Waltham, MA, USA ). After adding 500 µL of TRIzol to the larvae, 100 µL of chloroform was added, and the solution was mixed and incubated for 5 min. After centrifugation (12,000 rpm, 4 °C, 15 min), the upper phase was transferred to a new tube, and 500 µL of isopropanol was added. After a 10-min incubation period and another centrifugation step (14,000 rpm, 4 °C, 10 min), the supernatant was discarded. The pellet was washed with 1 mL of 70% ethanol and centrifuged (9500 rpm, 4 °C, 5 min). The supernatant was removed, and the pellet was air dried. The pellet was dissolved in 20 µL of DEPC-treated water and incubated on ice for 10 min. The concentration and purity of the total RNA were determined using a NanoDrop device (Thermo Fisher Scientific, Waltham, MA, USA). Reverse transcriptase reactions were used to synthesize cDNA from 2 µg of total RNA using an Advantage RT-PCR kit (Clontech, Palo Alto, CA, USA) following the manufacturer’s protocols. Test gene expression levels were normalized against β-actin levels in each zebrafish larva sample.

### 4.7. Western Blot Analysis

The zebrafish larvae were lysed with lysis buffer. Then, 30 µg of total protein was separated via SDS-polyacrylamide gel electrophoresis and transblotted onto nitrocellulose membranes, which were then blocked with 5% skim milk in phosphate-buffered saline containing 0.2% Tween 20. Immunoblotting analysis was performed as per the manufacturer’s instructions with the primary antibodies anti-tyrosinase-related protein 1 (TRP1), anti-tyrosinase-related protein 2 (TRP2), anti-microphthalmia-associated transcription factor (MITF), anti-extracellular signal-regulated kinase (ERK), anti-phospho-ERK, and anti-β-actin. The membranes were incubated with the primary antibodies at a dilution of 1:2000 and then washed and incubated with horseradish peroxidase-conjugated secondary antibody.

### 4.8. Fontana-Masson Silver Staining

We performed Fontana-Masson silver staining to visualize melanin in the zebrafish larvae using a previously described method [36]. Formalin-fixed and paraffin sections of larvae were processed on slides and stained with silver nitrate (Kojima Chemical, Kashiwabara, Japan) and washed. Then, the slides were fixed and washed. Next, the slides were stained with nuclear fast red solution (Sigma Aldrich, Saint Louis, MO, USA) and washed. Finally, after dehydration with 95% and 100% ethanol, the slides were washed twice with xylene (Duksan, Seoul, Korea).

### 4.9. Statistical analysis

Data were analyzed using one-way analysis of variance (ANOVA) and student *t*-test. Differences between means were considered significant at *p* < 0.05 (* *p* < 0.05, ** *p* < 0.01). Graphical representations were produced using Sigmaplot 2001 (Systat Software, San Jose, CA, USA).

## 5. Conclusions

In the present study, we found that RBM induced pigmentation via ERK phosphorylation and the ERK signaling pathway. Although many further studies are needed to determine the efficacy and safety of RBM, the results of the present study suggest that RBM may be used to improve symptoms of hypopigmentary skin diseases. These data indicate that RBM possesses melanogenic activity and acts via the ERK pathway. Our results suggest that RBM may be useful as a cosmeceutical tanning agent, such as in the treatment of gray hair caused by reduced melanin synthesis in the hair follicle. Our future studies will focus on which compounds in the RBM promote pigmentation and RBM-induced pigmentation in a human hair follicle organ culture model.

## Figures and Tables

**Table 1:** Chemical composition of rice bran ash mineral extract (RBM).

Element	Si	Ca	Mg	K	Na	P
mg/kg (solution)	180	5	17	2930	113	300
